# Impact of Patient Characteristics on Treatment Outcomes in Symptomatic Venous Thromboembolism: Results of HOKUSAI-VTE Randomized Trial Analysis

**DOI:** 10.1055/s-0040-1716496

**Published:** 2020-09-23

**Authors:** Ben van Hout, Emma Hawe, Alexander T. Cohen

**Affiliations:** 1 Health Economics and Decision Science Division, University of Sheffield, Sheffield, United Kingdom; 2 Data Analytics and Design Strategy Division, RTI-Health Solutions, Manchester, United Kingdom; 3 Department of Haematology, Guy's and St Thomas' NHS Foundation Trust, London, United Kingdom

**Keywords:** venous thrombosis, edoxaban, warfarin, creatinine, effect modification, age

## Abstract

**Introduction** In patients with venous thromboembolism (VTE), direct oral anticoagulants (DOACs) such as edoxaban, apixaban, dabigatran, and rivaroxaban are more convenient, safer, and just as effective as vitamin K antagonists (VKAs). Limited information is known about the effects of patient characteristics on VTE efficacy and safety of DOACs compared with VKAs, without appropriate effect modifier adjustment comparisons of DOACs may be biased. This study considers the effect of variables that can modify the efficacy and safety of edoxaban and warfarin, using patient-level data.

**Materials and Methods** The primary efficacy and safety outcomes in the HOKUSAI-VTE study were VTE recurrence and clinically relevant bleeding, respectively. Potential effect modifiers were age, creatinine clearance, and weight. The relationship between the percentage of time in international normalized ratio (INR) control and outcomes were considered for the warfarin arm. Univariate and multivariate regression were performed for each patient characteristic.

**Results** The relationship between treatment and VTE recurrence differed by age (interaction *p* = 0.007) and by creatinine clearance ( *p* = 0.05). VTE recurrence differed by age for patients in the warfarin arm but not for those in the edoxaban arm and differed by INR control in the warfarin arm ( *p* < 0.005). A stronger relationship between creatinine clearance and clinically relevant bleeding was found in the warfarin arm than in the edoxaban arm ( *p* = 0.04). Clinically relevant bleeding differed by the percentage of time in INR control in the warfarin arm ( *p* < 0.005). Age appeared to be a more important effect modifier than creatinine clearance in patients with VTE.

**Discussion** The finding that efficacy in older patients was greater for those taking edoxaban than for those taking warfarin in the HOKUSAI-VTE study needs further investigation. Modification of the treatment effect by age for those taking warfarin might bias estimates of comparative effectiveness among DOACs if VKAs are the reference treatment.

## Introduction

Venous thromboembolism (VTE) is a major cause of morbidity and mortality. In Europe, the estimated incidence rate of VTE is approximately 130 per 100,000 patient-years, and therefore between 600,000 to over 1 million VTE events or deaths occur annually. 1 2 Early diagnosis and treatment often lead to recovery, but VTE recurrences and long-term complications, such as postthrombotic syndrome and chronic thromboembolic pulmonary hypertension, may occur.

For over 60 years, the cornerstone of treatment for VTE has been vitamin K antagonists (VKAs) such as warfarin. Treatment may be for short periods (3 months) or for longer periods (6 months to long term) to prevent VTE recurrences. However, VKAs decrease the clotting tendency of blood, and their effect must be monitored carefully with frequent blood tests and dose adaptations. Underdosing leads to increased risk of blood clots and excessive dosing to increased risk of bleeding.

Previous studies have shown that the risks of VTE and pulmonary embolism (PE) are four to six times higher in patients over 70 years old than in younger patients. 3 In addition, age has been found to increase the risk of bleeding related to anticoagulant use. The relationship between renal clearance and age is well known, with renal clearance reduced in proportion to increasing age.

In the past 5 to 10 years, new direct oral anticoagulants (DOACs), such as apixaban, dabigatran, edoxaban, and rivaroxaban, have been demonstrated to be more convenient and have a better safety profile than VKAs, with a proven effectiveness at preventing VTE recurrence and long-term complications. 4 5 6 7

Many patients and clinicians prefer a once-daily treatment regimen, which leads to a choice between two DOACs, rivaroxaban and edoxaban; however, no head-to-head, randomized controlled trial data are available.

The efficacy and safety of rivaroxaban versus VKAs has been investigated in the EINSTEIN studies, addressing deep vein thrombosis (DVT) and PE separately. 5 6 The efficacy and safety of edoxaban versus warfarin has been investigated in one study, the HOKUSAI-VTE study, which included both DVT and PE patients. 8 These studies revealed no notable differences in the overall results for both drugs; both studies showed similar efficacy and improved safety of rivaroxaban and edoxaban compared with VKA. However, the trials differed in terms of both their populations and design. Differences in study designs may be of particular importance in the presence of potential effect modifiers, such as age, creatinine clearance, and weight. Additionally, the level of control in the warfarin arm of the studies differed.

Previous meta-analysis has suggested increased efficacy of DOACs compared with VKAs in patients with low creatinine clearance compared with patients with high creatinine clearance, based on published aggregate data for subgroups. 3

This study aimed to estimate the impact on treatment outcomes of possible effect modifiers in patients with VTE, using patient-level data from the HOKUSAI-VTE study. When treatments are directly compared in real-world studies, patient characteristics that impact outcomes may differ between treatment arms. To ensure unbiased comparative efficacy and safety estimates, researchers need to be aware of these characteristics and use approaches that can adjust and minimize differences in effect modifiers between the treatment arms. For cases where there is an absence of head-to-head trials, indirect comparisons or network meta-analyses, which rely on the assumptions of homogeneity of study designs and trial populations, are often performed. However, in the presence of effect modification by patient characteristics or study design differences, especially in treatment patterns for the common comparator, the conclusions of network meta-analysis can be biased. Sophisticated methods are now available to generate indirect comparisons that can be made across different studies where patient-level data are available from one study and aggregate data are available from another study. 9 These approaches allow for the studies to be matched to adjust for differences in study design and effect modifiers. Per the Decision Support Unit guidelines of the United Kingdom's National Institute for Health and Clinical Excellence, 10 it is important to adjust for all variables that modify treatment effect of the new medications or the common comparator. Further, for comparisons of studies where effect modifiers differ between treatment arms, approaches such as propensity score weights are possible to adjust for the effect modifiers.

## Materials and Methods

### Study Populations

The efficacy and safety of edoxaban versus VKA were investigated in the HOKUSAI-VTE study that included 4,921 DVT and 3,319 PE patients enrolled at 439 centers in 37 countries between January 2010 and October 2012. 8 Randomization was performed with the use of an interactive Web-based system, with stratification according to the qualifying diagnosis (DVT or PE), presence or absence of temporary risk factors, and the dose of edoxaban. The HOKUSAI-VTE study was a randomized, double-blind study that compared heparin followed by edoxaban with heparin followed by warfarin in patients objectively diagnosed with acute symptomatic DVT involving the popliteal, femoral, or iliac veins or with acute, symptomatic PE with or without DVT. 8 Patients received the study drug for 3 to 12 months; the duration was determined by the treating physician and based on the patient's clinical features. The primary efficacy outcome was recurrent symptomatic VTE, and the principal safety outcome was major or clinically relevant nonmajor bleeding. Reported analysis of pooled data for both DVT and PE patients found a nonsignificant lower proportion of recurrent VTE (3.2% vs. 3.5%) for patients receiving edoxaban than for patients receiving warfarin. Significantly lower, clinically relevant bleedings (8.5% vs. 10.3%) were found in the edoxaban arm than in the warfarin arm. In this study, patient-level data for the modified intention-to-treat population (all randomized subjects who received at least one dose of the study drug) from the HOKUSAI-VTE study were analyzed for effect modifiers based on patient characteristics or study design.

### Effect Modifiers

Patient-level data from the HOKUSAI-VTE study (edoxaban vs. warfarin) were analyzed to identify patient characteristics at baseline that may be effect modifiers. Analyses were performed for the pooled DVT and PE groups, and further analyses were performed separately for those with PE and for those with DVT only (i.e., those with DVT and not PE). Patient characteristics considered to be potential effect modifiers were age, creatinine clearance, and weight. Creatinine clearance was calculated using the Cockcroft and Gault equation, which includes patients' age, weight, and gender. To assess the effect of a failing kidney/poor kidney function on top of the effect of age and weight, predicted minus observed creatinine clearance was calculated. Predicted minus observed creatinine clearance was calculated as the difference between predicted creatinine clearance from a linear regression model, which included age and weight as covariates, and observed creatinine clearance. Study design was considered only for the warfarin group as time in international normalized ratio (INR) control; within the study, the dose of warfarin was adjusted to maintain the INR between 2.0 and 3.0.

## Statistical Methods

The distribution of age, creatinine clearance, and body weight were considered through histograms. The relationship between efficacy (i.e., VTE recurrence) and patient characteristics was first addressed graphically by way of a scatterplot picturing event rates in 5% deciles (20 groups selected to have equal numbers of patients within each group, with sufficient patient numbers in each group to enable meaningful comparisons across the age groups). The plots were generated for age, creatinine clearance, and body weight. For each potential effect modifier of interest, a plot was generated for both treatment groups together (bottom-left quadrant) as well as separately for the edoxaban (top-right quadrant) and warfarin (bottom-right quadrant) treatment groups. A logistic model also was fit to each group and overlaid on the scatterplots; efficacy or safety outcomes were included as the dependent variable in the logistic models, and patient characteristics were included (as continuous variables) as the predictor within the model. The logistic models were run for both treatment arms together and for each of the treatment arms separately to align with the presented scatterplots. This analysis provided an initial assessment of the relationship between the potential effect modifiers and the outcomes of interest overall and in each treatment arm.

Univariate regression models also were performed to assess the statistical significance of effect modification by including an interaction term between treatment and each effect modifier of interest, age, creatinine clearance, and body weight. Predicted minus observed creatinine clearance was also considered as an effect modifier. To assess which of the effect modifiers was more predictive of changes in the outcomes, we performed a multivariate analysis. All potential effect modifiers were considered in a single model along with planned treatment duration (considered as a categorical variable for 3, 6, and 12 months of planned duration), and stepwise reduction of the model was performed to assess which effect modifiers remained significant predictors after adjusting for other covariates and interaction terms. Cox proportional hazards regression also was performed to consider whether age, creatinine clearance, or weight were treatment effect modifiers when simultaneously considering the timing of the event (VTE recurrence and clinically relevant bleeding) and appropriate censoring.

It is well known that INR control is a crucial predictor of events in the warfarin arm; as such, any relationship between events and baseline characteristics may be indirectly caused by a relationship between those baseline characteristics and therapeutic time in INR control. To explore this, we examined the correlations between the various baseline variables and INR control through a series of regression models, overlaid against scatterplots of the outcome variable by deciles of the predictor variables.

## Results

### Demographics

**Fig. 1:**
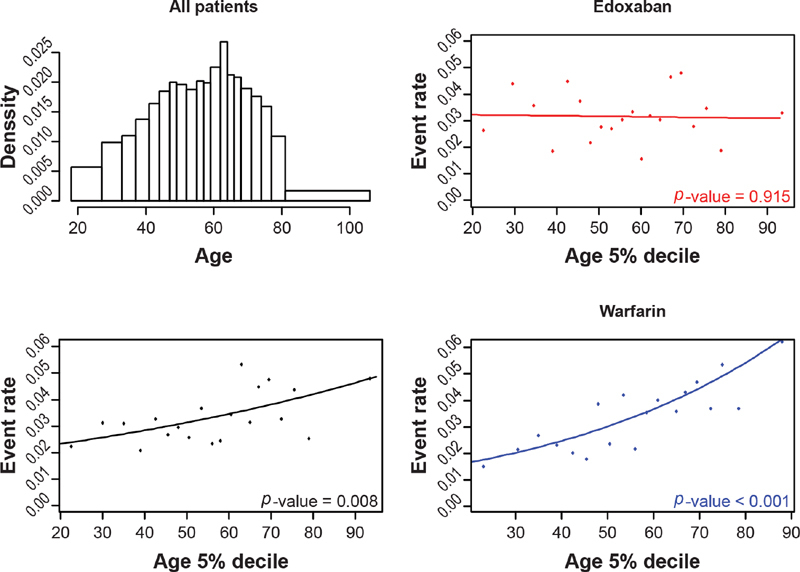
The distribution of age and the relationship between VTE recurrence and age, by treatment arm. VTE, venous thromboembolism. Note: The top-left quadrant in this figure shows the age distribution, the bottom-left quadrant shows the relationship between the incidence of recurrent VTE and age in all patients, and the two graphs on the right-hand side show recurrent VTE according to treatment.

The mean age was 55.7 years (standard deviation [SD], 16.3) in the edoxaban arm and 55.9 years (SD, 16.2) in the warfarin arm ( Table 1 ). The percentage of patients with creatinine clearance between 30 and 50 mL/min, inclusive, was 6.5% in the edoxaban arm and 6.6% in the warfarin arm. Of the patients in the edoxaban arm, 12.7% had a weight ≤ 60 kg compared with 12.6% in the warfarin arm; further, the proportion of those with high weight (> 100 kg) was similar between the two arms: 14.8% (edoxaban) and 15.9% (warfarin). Among patients receiving warfarin, the INR was in the therapeutic range for 63.5% of the time, above 3.0 for 17.6% of the time, and below 2.0 for 18.9% of the time. The distribution of the effect modifiers in all patients is shown in Figs. 1 2 3 .

**Fig. 2:**
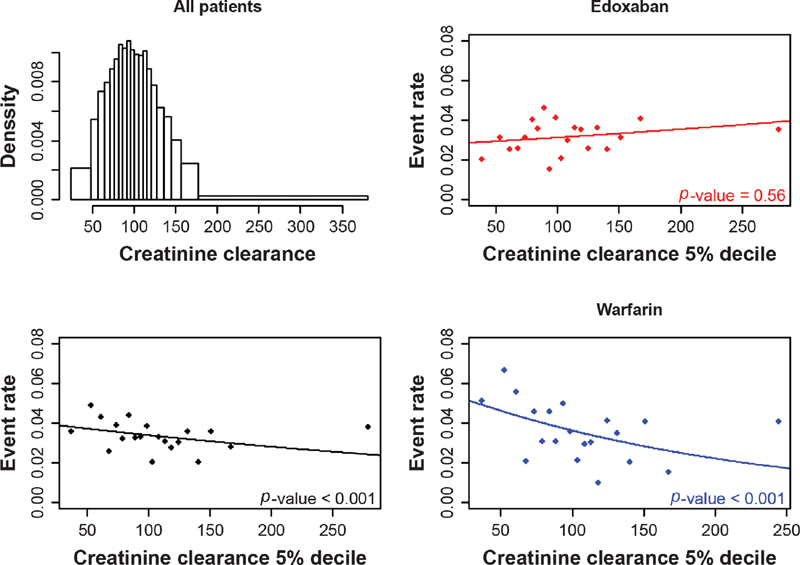
The distribution of creatinine clearance and the relationship between VTE recurrence and creatinine clearance, overall and by treatment arm. VTE, venous thromboembolism.

**Fig. 3:**
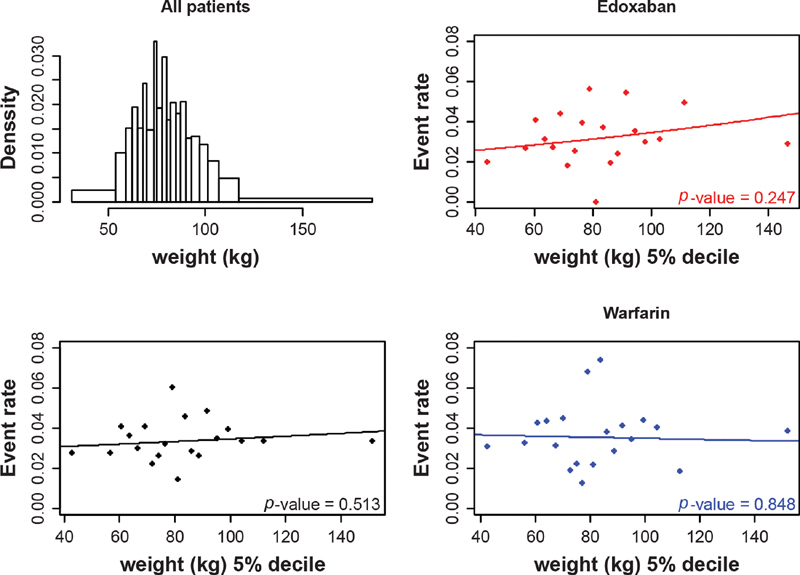
The distribution of body weight and the relationship between VTE recurrence and body weight, overall and by treatment arm. VTE, venous thromboembolism.

**Table 1:** Selected baseline characteristics in HOKUSAI-VTE study a

	All patients		Patients with DVT only		Patients with PE	
	Edoxaban ( *N* = 4,118)	Warfarin ( *N* = 4,122)	Edoxaban ( *N* = 2,468)	Warfarin ( *N* = 2,453)	Edoxaban ( *N* = 1,650)	Warfarin ( *N* = 1,669)
Age, mean (SD), y	55.7 (16.3)	55.9 (16.2)	54.7 (16.0)	54.9 (15.9)	57.1 (16.6)	57.4 (16.5)
Age ≥ 75 y, *n* (%)	560 (13.6)	544 (13.2)	282 (11.4)	273 (11.1)	278 (16.8)	271 (16.2)
Creatinine clearance: 30–50 mL/min, *n* (%)	268 (6.5)	273 (6.6)	152 (6.2)	153 (6.2)	116 (7.0)	120 (7.2)
Weight, *n* (%)						
< 60 kg	524 (12.7)	519 (12.6)	320 (13.0)	304 (12.4)	204 (12.4)	215 (12.9)
> 100 kg	611 (14.8)	654 (15.9)	360 (14.6)	379 (15.5)	251 (15.2)	275 (16.5)
Percentage of time:	—		—		—	
In INR control		63.62		62.63		65.11
INR above 3.0		16.54		17.09		15.70
INR below 2.0		19.84		20.27		19.19

Abbreviations: DVT, deep vein thrombosis; INR, international normalized ratio; PE, pulmonary embolism; SD, standard deviation.

a Results are based on the modified intention to treat population.

#### Exploring Possible Effect Modifiers

The effect of age on VTE recurrence is illustrated in Fig. 1 . As expected, VTE recurrence was found to differ according to age (overall *p* = 0.01 from logistic regression), with higher event rates observed as age increased from baseline. However, when only those patients on edoxaban was considered, VTE recurrence did not differ with age ( *p* = 0.915); whereas for the subset of patients taking warfarin, recurrence increased with age ( *p* < 0.005).

Age had almost no effect on VTE recurrence in the edoxaban arm, but VTE recurrence increased with increasing age in the warfarin arm ( Fig. 1 ; Supplementary Fig. S1 ). The estimates of the univariate logistic regressions for interactions between treatment and the effect modifiers of age, creatinine clearance, weight, and INR control (limited to the warfarin arm) are shown in Table 2 . The effect of treatment on VTE recurrence significantly differed by age (interaction *p* = 0.007), with the odds of having an event increasing by 11% for every 5-year increase in age. Based on these results, the odds of having a recurrent VTE were equal between ages 52 and 53 years. Below age 52 years, the odds were lower for the warfarin group; above age 53 years, the odds were in favor of edoxaban. After adjustment was made for treatment duration, the significant interaction between age and treatment was still observed.

**Table 2:** Univariate analysis of efficacy and safety event rates

	VTE recurrence			Clinically relevant bleeding		
Age	Estimate	Standard error	Pr(>| *z* |)	Estimate	Standard error	Pr(>| *z* |)
(Intercept)	–3.391	0.316	< 0.005	–3.040	0.212	< 0.005
Age	–0.001	0.005	0.915	0.012	0.004	0.001
Treatment	–1.106	0.467	0.018	0.013	0.291	0.965
Age × treatment	0.021	0.008	0.007	0.003	0.005	0.486
Predicted minus observed creatinine clearance a	Estimate	Standard error	Pr(>| *z* |)	Estimate	Standard error	Pr(>| *z* |)
(Intercept)	–3.424	0.092	< 0.005	–2.383	0.058	< 0.005
Predicted minus observed creatinine clearance	0.000	0.004	0.996	–0.005	0.002	0.018
Treatment	0.144	0.126	0.251	0.209	0.078	0.008
Predicted minus observed creatinine clearance × treatment	0.000	0.005	0.993	0.006	0.003	0.074
Creatinine clearance b	Estimate	Standard error	Pr(>| *z* |)	Estimate	Standard error	Pr(>| *z* |)
(Intercept)	–3.563	0.255	< 0.005	–2.305	0.160	< 0.005
Creatinine clearance	0.001	0.002	0.560	–0.001	0.001	0.649
Treatment	0.792	0.351	0.024	0.627	0.219	0.004
Creatinine clearance × treatment	–0.006	0.003	0.047	–0.004	0.002	0.036
Weight	Estimate	Standard error	Pr(>| *z* |)	Estimate	Standard error	Pr(>| *z* |)
(Intercept)	–3.844	0.380	< 0.005	–2.457	0.241	< 2e-16
Weight	0.005	0.004	0.247	0.001	0.003	0.741
Treatment	0.609	0.521	0.243	0.633	0.326	0.053
Treatment × weight	–0.006	0.006	0.333	–0.005	0.004	0.186
INR control	Estimate	Standard error	Pr(>| *z* |)	Estimate	Standard error	Pr(>| *z* |)
(Intercept)	–2.602	0.262	< 0.005	–1.497	0.161	< 0.005
% of time in INR control	–0.013	0.004	< 0.005	–0.012	0.003	< 0.005

Abbreviations: INR, international normalized ratio; VTE, venous thromboembolism.

Note: Edoxaban is the reference group in the models, and warfarin is coded as 1.

a Predicted minus observed creatinine clearance was calculated as the difference between predicted creatinine clearance from a linear regression model, with creatinine clearance as the dependent variable and age and weight as covariates, and observed creatinine clearance. Predicted minus observed creatinine clearance is a marker of the effect of a failing kidney/poor kidney function on top of the effect of age and weight.

b The variable creatinine clearance is based on the Cockcroft and Gault equation, which includes patients age, weight and gender. By using the coefficients from the model, it is possible to estimate the impact of age on VTE recurrence in patients in the warfarin arm and patients in the edoxaban arm. For those in the edoxaban arm, a 5-year increase in age is associated with an increase in the odds of having an event by: (include calc) = 11%. The odds of having a recurrent VTE for edoxaban is below that of warfarin for patients above the age of 53: exp (–3.391 + 53 × –0.001) < exp (–3.391 + 53 × –0.001 + 1 × –1.106 + 53 × 0.021). For patients below the age of 52 years, the odds are lower for the warfarin group than for the edoxaban group: exp (–3.391 + 52 × –0.001) < exp (–3.391 + 52 × –0.001 + 1 × –1.106 + 52 × 0.021).

Based on the separate regression models as creatinine clearance increased, VTE recurrence decreased for the warfarin arm but increased for the edoxaban arm ( Fig. 2 ). In line with the separate regression models, a statistically significant interaction was observed between treatment and creatinine clearance, with VTE recurrence in the univariate analysis ( *p* = 0.047; Table 2 ; Supplementary Fig. S2 ). When the difference between predicted creatinine clearance based on age and weight and observed creatinine clearance was considered, no significant interaction with treatment was observed ( *p* = 0.93).

No significant interaction was found between weight and treatment on VTE recurrence ( *p* = 0.33; Table 2 ; Fig. 3 ), and no statistically significant relationship was observed between weight and VTE recurrence in either the edoxaban or the warfarin arm ( *p* = 0.25 and *p* = 0.80, respectively). A decrease in VTE events was seen for patients who spent more time in INR control ( *p* = 0.001; the coefficient from the logistic regression model was –0.013).

In the multivariate model with VTE recurrence as the dependent variable which considered the variables weight, duration of treatment, age, INR control, creatinine clearance (uncorrected), and interaction terms, only the interaction between treatment and age remained significant within the model following stepwise reduction. Similarly, in a model initially considering predicted minus observed creatinine clearance following stepwise reduction, the interaction between age and treatment remained the only significant interaction term in the model.

Analysis considering DVT and PE patients separately is reported in the Supplementary Material . The interaction between age and treatment was seen in the DVT group only; VTE recurrence was higher with increasing age in the warfarin group than in the edoxaban group, and the INR relationship was most pronounced in the PE group ( Supplementary Tables S1 and S2 ). The results of the Cox proportional hazards models were in alignment with the findings from the logistic regression models ( Supplementary Table S3 ).

**Fig. 4:**
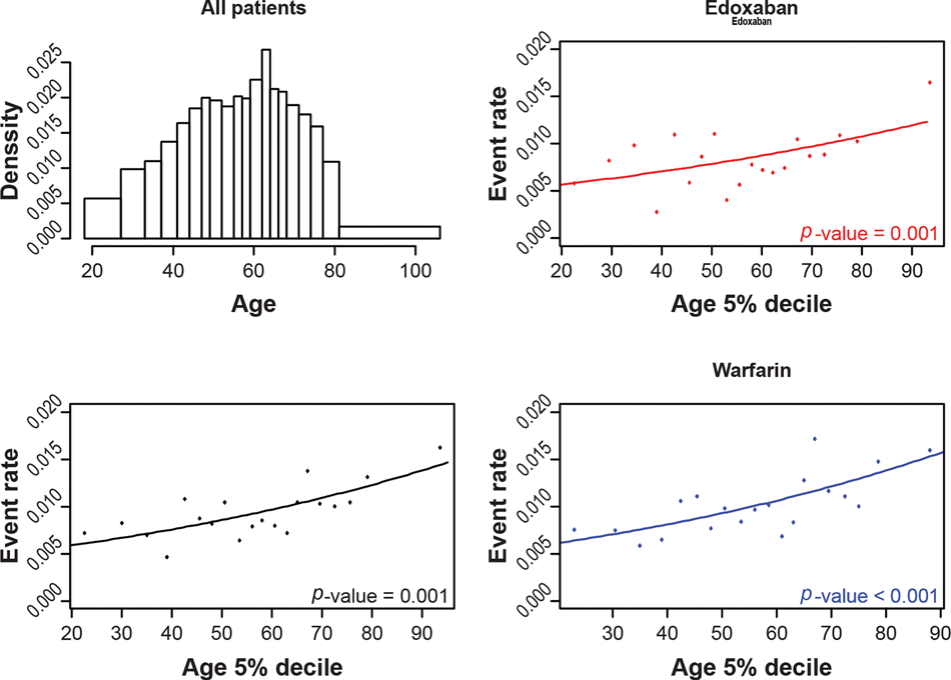
The relationship between clinically relevant bleeding and age, overall and by treatment arm.

**Fig. 5:**
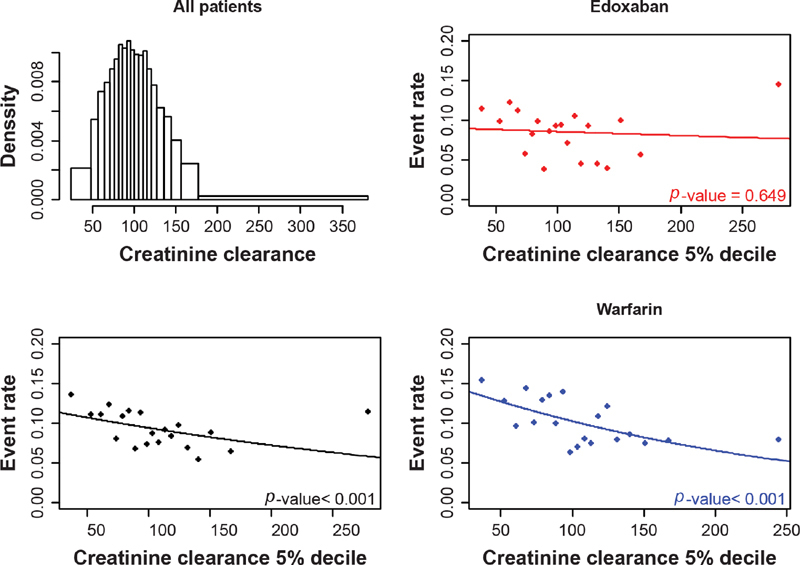
The relationship between clinically relevant bleeding and creatinine clearance, overall and by treatment arm.

**Fig. 6:**
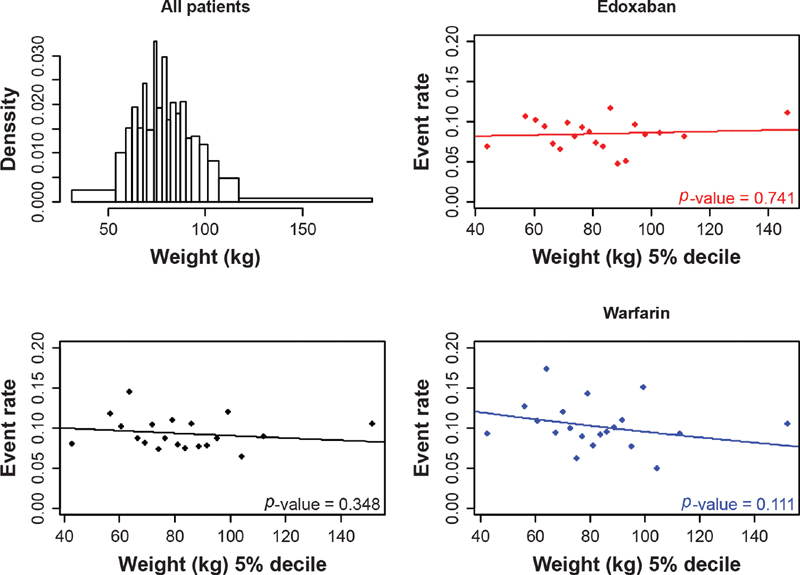
The relationship between clinically relevant bleeding and body weight, overall and by treatment arm.

As age increased, there was an increase in clinically relevant bleeding, and this relationship appeared similar across both arms ( Fig. 4 ). For safety, the relationship between creatinine clearance and clinically relevant bleeds differed by treatment arm ( Table 2 ; Fig. 5 ). For warfarin, a decrease in clinically relevant bleeding was observed as creatinine clearance increased; a weaker negative relationship was observed in the edoxaban arm, but this did not reach significance. Although a statistically significant interaction between treatment and creatinine clearance on clinically relevant bleeding was observed when considering VTE and PE combined ( Supplementary Fig. S3 ), it was not observed when considering VTE or PE separately ( Supplementary Tables S1 and S2 ). There did not appear to be a clear relationship between weight and the number of bleeds in patients in either the edoxaban arm or the warfarin arm ( Table 2 ; Fig. 6 ). A significant relationship was found between INR control and bleeding events for patients taking warfarin, with decreased clinically relevant bleeding observed as percentage of time in INR control increased ( Table 2 ).

**Fig. 7:**
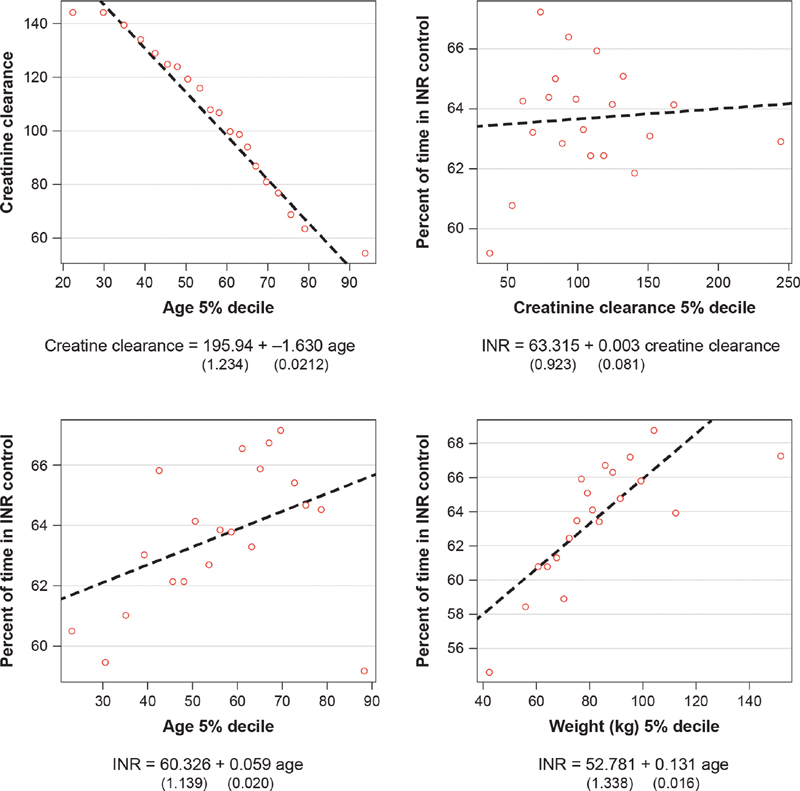
relationship between age, INR control, and creatinine clearance. INR, international normalized ratio.

As seen in Fig. 7 , creatinine clearance was strongly correlated with age, with creatinine clearance decreasing as age increased. The upper left-hand quadrant of Fig. 7 shows the average creatinine clearance of 20 equally sized age groups and indicates a strong relationship between age and creatinine clearance. The lower-left plane of Fig. 7 indicates a significant positive relationship between age and percentage of time in INR control, which suggests that the negative relationships between age and events are not linked through INR control. Contrarily, a nonsignificant positive relationship was found between creatinine clearance and INR control. Additionally, there was a strong relationship between weight and time in INR control.

## Discussion

We analyzed the effects of a selection of risk factors, using patient-level data from the HOKUSAI-VTE study. 7 It was assumed a priori that the most important variables to consider would be age, creatinine clearance, and weight, together with a distinction between patients who were to be on treatment for 3, 6, or 12 months. Analysis showed a clear increase in VTE recurrence rate with respect to age in the warfarin patients but no such effect in the edoxaban patients. Assuming a linear relationship in the log-odds, it is estimated that the cutoff point at which treatment with edoxaban becomes more effective than warfarin is approximately 52 years of age. A similar relationship was seen in a pooled analysis of the RE-COVER studies, with the efficacy of dabigatran compared with warfarin being higher in older patients and lower in younger patients, 11 although this did not reach statistical significance, and no relationship was reported in published subgroup analysis of the same studies. 3 Based on a published subgroup analysis of patients older and younger than 75 years of age, the EINSTEIN studies also reported a relationship between events and age in the usual-care arm, although the EINSTEIN results seem less pronounced than that which may be expected. 5 12

A similar effect on treatment outcomes in the HOKUSAI-VTE trial was found with respect to creatinine clearance, but creatinine clearance is strongly related to age. Due to this interrelation (collinearity), it was difficult to ascertain whether the creatinine clearance or increasing age is associated with edoxaban being more effective. However, in models where creatinine clearance was considered with adjustment for age, the relationship between creatinine and VTE recurrence did not differ by treatment arm. The predicted minus observed creatinine clearance variable is a marker of kidney function that is above or below that expected based on a patient's age and weight. Since the interaction between treatment and predicted minus observed creatinine clearance was not significant, it indicates that a level of creatinine clearance that differs from that expected based on a patient's age and weight does not modify the effect of a treatment's efficacy and safety. Further, the multivariate analysis suggested that age was a stronger modifier than creatinine clearance on treatment efficacy and safety. This result may suggest that if the modifying effect of age on treatment efficacy and safety is considered in patients with VTE, it is not necessary to consider creatinine clearance. While a relationship between weight and events was assessed, no such significant relationship was observed in the HOKUSAI-VTE trial.

Age may be considered as a surrogate marker for several other factors that may influence the treatment efficacy and bleeding risk. In addition to the relationship between age and creatinine clearance, concomitant conditions and the type of venous disease may be anticipated to differ between older and younger patients. The relationship between age and other risk factors for VTE may be complex; for example, while aging and postmenopausal status have been found to enhance the risk of chemotherapy-induced VTE in women with breast cancer, the rate of cancer diagnosis in the first year after VTE onset has been found to be lower in the elderly compared with younger patients. 13 The number of factors with which age is related may be one explanation for age being a stronger treatment effect modifier than creatinine clearance in the HOKUSAI-VTE trial.

INR control did not explain the significant interaction found between age and treatment on efficacy in the warfarin arm. This outcome strengthens the relationship between increasing age and decreasing efficacy of warfarin. Additional analyses that adjusted for the planned treatment duration did not alter this finding. While age and creatinine clearance are strongly negatively correlated, and age and percentage of time in INR control are positively correlated, one would expect a significant negative relationship between creatinine clearance and INR control.

## Conclusion

Analysis showed a consistent rate of VTE recurrence with respect to age in the edoxaban patient, while warfarin patients had a clear increase in the HOKUSAI-VTE trial. Similarly, outcomes differed by creatinine clearance in the warfarin patients but not in the edoxaban patients. Age appeared to be a stronger predictor of efficacy and safety than creatinine clearance, although the two are highly correlated.

To compare DOACs based on data from different studies, it is important to consider differences in possible effect modifiers, including patient characteristics and study design. This analysis of data from the HOKUSAI-VTE study highlighted key potential effect modifiers of treatment effect, which may have implications for both direct comparisons between DOACs and VKAs and indirect comparisons between DOACs. Further research to confirm these findings would be of benefit, it would also be of interest to investigate these findings in cancer patients taking DOACs compared with VKAs.

## References

[1] Martinez C Cohen A T Bamber L Rietbrock S Epidemiology of first and recurrent venous thromboembolism: a population-based cohort study in patients without active cancer Thromb Haemost 2014 112 02 255 263 24695909 10.1160/TH13-09-0793

[2] VTE Impact Assessment Group in Europe (VITAE) Cohen A T Agnelli G Anderson F A Venous thromboembolism (VTE) in Europe. The number of VTE events and associated morbidity and mortality Thromb Haemost 2007 98 04 756 764 17938798 10.1160/TH07-03-0212

[3] Geldhof V Vandenbriele C Verhamme P Vanassche T Venous thromboembolism in the elderly: efficacy and safety of non-VKA oral anticoagulants Thromb J 2014 12 21 31 25650285 10.1186/1477-9560-12-21 PMC4314657

[4] AMPLIFY Investigators Agnelli G Buller H R Cohen A Oral apixaban for the treatment of acute venous thromboembolism N Engl J Med 2013 369 09 799 808 23808982 10.1056/NEJMoa1302507

[5] EINSTEIN Investigators Bauersachs R Berkowitz S D Brenner B Oral rivaroxaban for symptomatic venous thromboembolism N Engl J Med 2010 363 26 2499 2510 21128814 10.1056/NEJMoa1007903

[6] Buller H R Oral rivaroxaban for the treatment of symptomatic venous thromboembolism: a pooled analysis of the Einstein DVT and Einstein PE studies Blood 2012 120 21 2022535658

[7] RE-COVER Study Group Schulman S Kearon C Kakkar A K Dabigatran versus warfarin in the treatment of acute venous thromboembolism N Engl J Med 2009 361 24 2342 2352 19966341 10.1056/NEJMoa0906598

[8] Hokusai-VTE Investigators Büller H R Décousus H Grosso M A Edoxaban versus warfarin for the treatment of symptomatic venous thromboembolism N Engl J Med 2013 369 15 1406 1415 23991658 10.1056/NEJMoa1306638

[9] Signorovitch J E Sikirica V Erder M H Matching-adjusted indirect comparisons: a new tool for timely comparative effectiveness research Value Health 2012 15 06 940 947 22999145 10.1016/j.jval.2012.05.004

[10] NICE DSU Technical Support Document 18: Methods for population-adjusted indirect comparisons in submissions to NICE Phillippo D Ades T Dias S Palmer S Abrams K Welton N 2016. Accessed January 27, 2020 at:http://www.nicedsu.org.uk/wp-content/uploads/2017/05/Population-adjustment-TSD-FINAL.pdf

[11] RE-COVER II Trial Investigators Schulman S Kakkar A K Goldhaber S Z Treatment of acute venous thromboembolism with dabigatran or warfarin and pooled analysis Circulation 2014 129 07 764 772 24344086 10.1161/CIRCULATIONAHA.113.004450

[12] Mitchell A P Conway S E Rivaroxaban for treatment of venous thromboembolism in older adults Consult Pharm 2014 29 09 627 630 25203412 10.4140/TCP.n.2014.627

[13] Fimognari F L Repetto L Moro L Gianni W Incalzi R A Age, cancer and the risk of venous thromboembolism Crit Rev Oncol Hematol 2005 55 03 207 212 15979886 10.1016/j.critrevonc.2005.04.011

