# Supplementary material for: Impact of Patient Characteristics on Treatment Outcomes in Symptomatic Venous Thromboembolism: Results of HOKUSAI-VTE Randomized Trial Analysis
Source: TH Open. 2020 Sep 23;4(3):e245–54. doi: 10.1055/s-0040-1716496 (PMC7553796; doi:10.1055/s-0040-1716496)
Supplement: Supplementary file 1 — Supplementary Material [file 10-1055-s-0040-1716496-s200053.pdf]

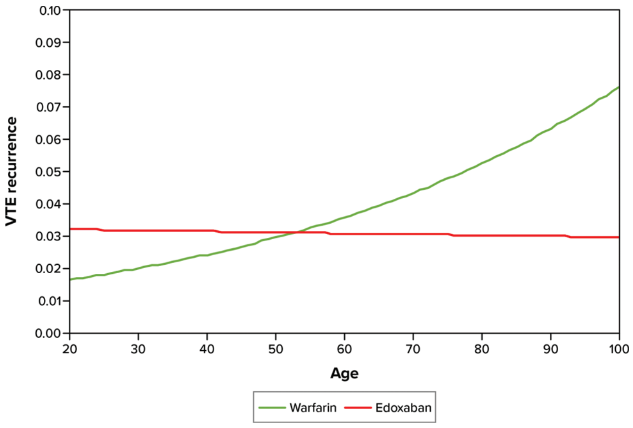

Supplementary Fig. S1. The relationship between age and VTE recurrence, by treatment arm, based on the univariate logistic regression model. VTE, venous thromboembolism.

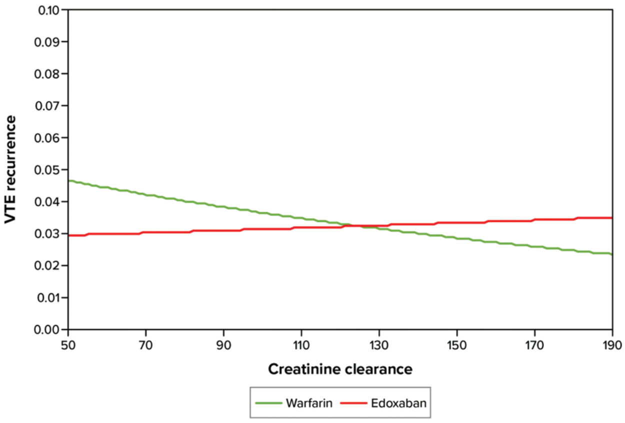

VTE = venous thromboembolism.

Supplementary Fig. S2. The Relationship Between Creatinine Clearance and VTE.

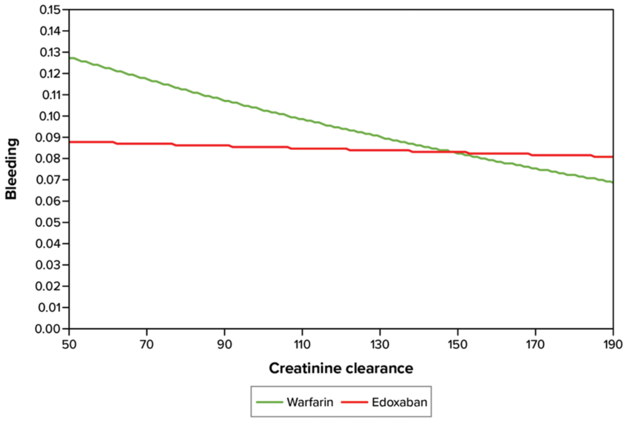

VTE = venous thromboembolism.

Supplementary Fig. S3. The Relationship Between Creatinine Clearance and Clinically.

Supplementary Table S1 Univariate analysis of efficacy and safety event rates for patients with DVT only

	VTE recurrence			Clinically relevant bleeding		
Age	**Estimate**	**Standard error**	**Pr(>|z|)**	**Estimate**	**Standard error**	**Pr(>|z|)**
(Intercept)	−2.98445	0.384459	<0.005	−3.32	0.294	<0.005
Age	−0.00694	0.006941	0.3173	0.0141	0.00492	0.00418
Treatment	−1.24782	0.584595	0.0328	0.278	0.395	0.48197
Age × treatment	0.022035	0.010066	0.0286	0.000088	0.00660	0.98941
Predicted minus observed creatinine clearance[a]	**Estimate**	**Standard error**	**Pr(>|z|)**	**Estimate**	**Standard error**	**Pr(>|z|)**
(Intercept)	−3.38746	0.115998	<0.005	−2.52546	0.078871	<0.005
Predicted minus observed creatinine clearance	0.001721	0.004994	0.73	−0.00457	0.003256	0.16022
Treatment	0.040561	0.163071	0.804	0.276761	0.105927	0.00898
Predicted minus observed creatinine clearance × treatment	0.000825	0.006976	0.906	0.00193	0.004345	0.65688
Creatinine clearance[b]	**Estimate**	**Standard error**	**Pr(>|z|)**	**Estimate**	**Standard error**	**Pr(>|z|)**
(Intercept)	−3.72093	0.33464	<0.005	−2.25874	0.226908	<0.005
Creatinine clearance	0.003077	0.002838	0.278	−0.00253	0.00207	0.222
Treatment	0.754132	0.467403	0.107	0.403248	0.30343	0.184
Creatinine clearance × treatment	−0.00672	0.004133	0.104	−0.00122	0.002774	0.66
Weight	**Estimate**	**Standard error**	**Pr(>|z|)**	**Estimate**	**Standard error**	**Pr(>|z|)**
(Intercept)	−4.09524	0.475976	<0.005	−2.29021	0.334744	<0.005
Weight	0.008913	0.005469	0.103	−0.00287	0.00405	0.478
Treatment	0.563384	0.677504	0.406	0.686559	0.449701	0.127
Treatment × weight	−0.00702	0.007869	0.372	−0.00497	0.005462	0.362
INR control	**Estimate**	**Standard error**	**Pr(>|z|)**	**Estimate**	**Standard error**	**Pr(>|z|)**
(Intercept)	−2.89608	0.347141	<0.005	−1.45956	0.201945	<0.005
% of time in INR control	−0.00903	0.005532	0.103	−0.01327	0.003261	<0.005

Abbreviations: DVT, deep vein thrombosis; INR, international normalized ratio; VTE, venous thromboembolism.
[a]Predicted minus observed creatinine clearance was calculated as the difference between predicted creatinine clearance from a linear regression model, with creatinine clearance as the dependent variable and age and weight as covariates, and observed creatinine clearance. Predicted minus observed creatinine clearance is a marker of the effect of a failing kidney/poor kidney function on top of the effect of age and weight.
[b]The variable creatinine clearance is based on the Cockcroft and Gault equation, which includes patients' age, weight, and gender.

Supplementary Table S2 Univariate analysis of efficacy and safety event rates for patients with PE

	VTE recurrence			Clinically relevant bleeding		
**Age**	**Estimate**	**Standard error**	**Pr(>|z|)**	**Estimate**	**Standard error**	**Pr(>|z|)**
(Intercept)	−4.18518	0.571371	<0.005	−2.61988	0.306095	<0.005
Age	0.011196	0.009204	0.224	0.00741	0.005021	0.14
Treatment	−0.63491	0.796741	0.426	−0.34197	0.434144	0.431
Age × treatment	0.015447	0.012532	0.218	0.00771	0.007031	0.273
Predicted minus observed creatinine clearance[a]	**Estimate**	**Standard error**	**Pr(>|z|)**	**Estimate**	**Standard error**	**Pr(>|z|)**
(Intercept)	−3.488	0.150361	<0.005	−2.19444	0.085267	<0.005
Predicted minus observed creatinine clearance	−0.00248	0.006005	0.68	−0.0062	0.003273	0.0583
Treatment	0.29904	0.198552	0.132	0.111894	0.117527	0.3411
Predicted minus observed creatinine clearance × treatment	−0.00117	0.008319	0.888	0.011029	0.004982	0.0269
Creatinine clearance[b]	**Estimate**	**Standard error**	**Pr(>|z|)**	**Estimate**	**Standard error**	**Pr(>|z|)**
(Intercept)	−3.33382	0.400125	<0.005	−2.31402	0.222237	<0.005
Creatinine clearance	−0.00149	0.003653	0.683	0.001316	0.001943	0.49821
Treatment	0.793483	0.537614	0.14	0.839438	0.314316	0.00757
Creatinine clearance × treatment	−0.00524	0.005132	0.307	−0.00741	0.002925	0.01133
Weight	**Estimate**	**Standard error**	**Pr(>|z|)**	**Estimate**	**Standard error**	**Pr(>|z|)**
(Intercept)	−3.4179	0.638454	<0.005	−2.56168	0.349123	<0.005
Weight	−0.0013	0.007575	0.863	0.004439	0.004046	0.273
Treatment	0.55474	0.829907	0.504	0.503803	0.474411	0.288
Treatment × weight	−0.00282	0.009898	0.776	−0.00458	0.005541	0.409
INR control	**Estimate**	**Standard error**	**Pr(>|z|)**	**Estimate**	**Standard error**	**Pr(>|z|)**
(Intercept)	−3.2874	0.23066	<0.005	−2.31808	0.151018	<0.005
% of time in INR control	0.002403	0.004614	0.603	0.00072	0.00312	0.818

Abbreviations: INR, international normalized ratio; PE, pulmonary embolism; VTE, venous thromboembolism.
[a]Predicted minus observed creatinine clearance was calculated as the difference between predicted creatinine clearance from a linear regression model, with creatinine clearance as the dependent variable and age and weight as covariates, and observed creatinine clearance. Predicted minus observed creatinine clearance is a marker of the effect of a failing kidney/poor kidney function on top of the effect of age and weight.
[b]The variable creatinine clearance is based on the Cockcroft and Gault equation, which includes patients age, weight and gender.

Supplementary Table S3 Cox proportional hazards models for efficacy and safety event rates

	VTE recurrence			Clinically relevant bleeding		
Age	**exp(coef)**	**se(coef)**	***p***	**Estimate**	**Standard error**	***p***
Age	1.000	0.005	0.932	1.012	0.003	<0.005
Treatment	0.334	0.461	0.017	0.995	0.284	0.986
Age × treatment	1.021	0.008	0.006	1.004	0.005	0.440
Predicted minus observed creatinine clearance[a]	**exp(coef)**	**se(coef)**	***p***	**Estimate**	**Standard error**	**Pr(>|*z*|)**
Predicted minus observed creatinine clearance	1.000	0.004	0.983	0.995	0.002	0.012
Treatment	1.150	0.123	0.259	1.227	0.075	0.006
Adjusted creatinine clearance × treatment	1.000	0.005	0.979	1.005	0.003	0.099
Predicted minus observed clearance[b]	**exp(coef)**	**se(coef)**	***p***	**Estimate**	**Standard error**	***p***
Creatinine clearance	1.001	0.002	0.572	0.999	0.001	0.427
Treatment	2.169	0.347	0.026	1.824	0.212	0.005
Creatinine clearance × treatment	0.994	0.003	0.049	0.996	0.002	0.040
Weight	**exp(coef)**	**se(coef)**	***p***	**Estimate**	**Standard error**	***p***
Weight	1.005	0.004	0.269	0.999	0.003	0.802
Treatment	1.776	0.513	0.263	1.749	0.315	0.076
Treatment × weight	0.994	0.006	0.357	0.996	0.004	0.254
INR control	**Estimate**	**Standard error**	***p***	**Estimate**	**Standard error**	***p***
% of time in INR control	1.002277	0.004509	0.614	0.985722	0.002521	<0.005

Abbreviations: INR, international normalized ratio; VTE, venous thromboembolism·
[a]Predicted minus observed creatinine clearance was calculated as the difference between predicted creatinine clearance from a linear regression model, with creatinine clearance as the dependent variable and age and weight as covariates, and observed creatinine clearance. Predicted minus observed creatinine clearance is a marker of the effect of a failing kidney/poor kidney function on top of the effect of age and weight.
[b]The variable creatinine clearance is based on the Cockcroft and Gault equation, which includes patients age, weight and gender.
